# Calibrated small-caliber-tip transparent hood enables rescue of discontinued colorectal endoscopic submucosal dissection

**DOI:** 10.1055/a-2751-0273

**Published:** 2025-12-15

**Authors:** Yuichiro Mikuriya, Kazuo Shiotsuki, Nobuhisa Minakata, Kohei Takizawa, Shin Maeda

**Affiliations:** 1 91321 Department of Gastroenterology, Kanagawa Cancer Center, Yokohama, Japan; 2 26438 Department of Gastroenterology, Yokohama City University Graduate School of Medicine, Yokohama, Japan

Colorectal endoscopic submucosal dissection (ESD) is becoming a standard treatment worldwide. Although this procedure is generally safe and effective, it can be technically challenging in certain situations, particularly in the case of severe submucosal fibrosis 1 2 . Although various technical innovations have improved outcomes for fibrotic lesions 3 4 , challenging cases require additional strategies. A calibrated small-caliber-tip transparent hood (CAST hood; TOP, Tokyo, Japan) features a tapered tip design that facilitates submucosal entry into fibrotic areas 5 . Herein, we present a case of severe fibrosis in which a CAST hood was employed in the rescue of discontinued colorectal ESD, including practical technical tips for daily practice.

A 74-year-old man was referred to our hospital after incomplete ESD of a large protruding lesion in the rectosigmoid colon ( Fig. 1 ). During the initial procedure at another hospital, approximately 75% of the circumferential incision and a partial dissection were performed before the procedure was discontinued. Rescue ESD was attempted using an EG-840T endoscope (Fujifilm, Tokyo, Japan), a 2.0 mm Proknife (Boston Scientific Japan K.K., Tokyo, Japan), and a VIO3 generator (Tübingen, Germany; Video 1 ). Scarring from the prior intervention suggested severe submucosal fibrosis. During dissection with a standard small-caliber-tip transparent hood (DH-33GR; Fujifilm, Tokyo, Japan) using the underwater technique, repeated submucosal entry into fibrotic areas became increasingly difficult due to the narrow working space. Therefore, we switched to a CAST hood, which allowed smooth penetration into narrow spaces and tissue elevation, facilitating visualization and adequate tissue tension ( Fig. 2 ).

**Fig. 1:**
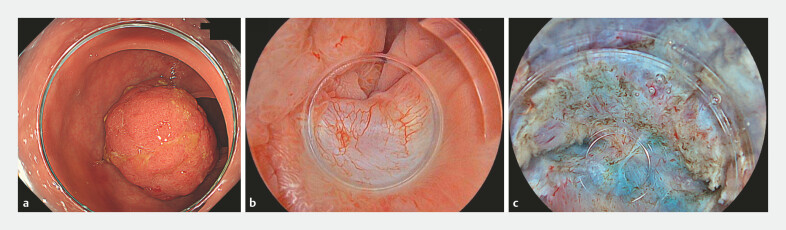
Endoscopic findings of the lesion and severe fibrosis from prior incomplete ESD. **a** A 50-mm Paris type 0-I lesion in the rectosigmoid colon. **b** Scarring at the site of the prior incomplete ESD. **c** Severe submucosal fibrosis observed during submucosal dissection. ESD, endoscopic submucosal dissection.

The use of the CAST hood for rescue of discontinued colorectal endoscopic submucosal dissection in the case of severe fibrosis: Practical tips. CAST, calibrated small-caliber-tip transparent.Video 1

**Fig. 2:**
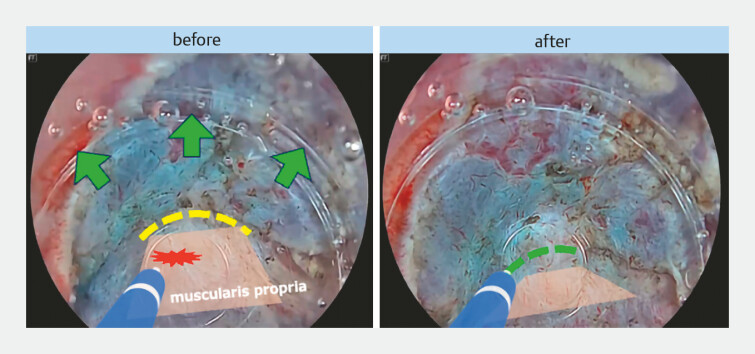
The CAST hood enabling submucosal entry in fibrotic areas. The hood with a tapered tip design enabled penetration into narrow spaces and tissue elevation. CAST, calibrated small-caliber-tip transparent.

En bloc and R0 resections were performed without complications. Pathological findings revealed intramucosal adenocarcinoma with negative margins.

The CAST hood can serve as a useful rescue option when standard techniques are challenging in patients with fibrosis.

Endoscopy_UCTN_Code_TTT_1AQ_2AD_3AD

## Notes

Endoscopy E-Videos https://eref.thieme.de/e-videos
*E-Videos* is an open access online section of the journal *Endoscopy* , reporting on interesting cases and new techniques in gastroenterological endoscopy. All papers include a high-quality video and are published with a Creative Commons CC-BY license. Endoscopy E-Videos qualify for HINARI discounts and waivers and eligibility is automatically checked during the submission process. We grant 100% waivers to articles whose corresponding authors are based in Group A countries and 50% waivers to those who are based in Group B countries as classified by Research4Life (see: https://www.research4life.org/access/eligibility/ ). This section has its own submission website at https://mc.manuscriptcentral.com/e-videos .
